# Indoor development of *Aedes aegypti* in Germany, 2016

**DOI:** 10.2807/1560-7917.ES.2016.21.47.30407

**Published:** 2016-11-24

**Authors:** Helge Kampen, Stephanie Jansen, Jonas Schmidt-Chanasit, Doreen Walther

**Affiliations:** 1 Friedrich-Loeffler-Institut, Federal Research Institute for Animal Health, Greifswald - Insel Riems, Germany; 2 Bernhard-Nocht-Institute for Tropical Medicine, Hamburg, Germany; 3 Leibniz-Centre for Agricultural Landscape Research, Muencheberg, Germany

**Keywords:** *Aedes aegypti*, Germany, invasive mosquito, public health, surveillance, vector

## Abstract

In spring 2016, a German traveller returning from Martinique cultivated imported plant offsets in her home, and accidentally bred *Aedes aegypti*. Thirteen adult mosquito specimens submitted for identification and the traveller were tested for Zika, dengue and chikungunya virus infections, with negative results. The detection of *Ae. aegypti* by the ‘Mueckenatlas’ project demonstrates the value of this passive surveillance scheme for potential public health threats posed by invasive mosquitoes in Germany.

In this report we present the accidental introduction by a traveller from the Caribbean into Germany, of *Aedes aegypti* eggs attached to plants, and further indoor development of adult mosquitoes from larvae hatched from these eggs in the traveller’s household in Germany. The mosquitoes were collected and killed, and some of them were subsequently tested for Zika, dengue and chikungunya viruses. The traveller was also tested for infections with these viruses.

## The event

In late March 2016, a German traveller who had visited her son on Martinique, brought home with her offsets of three exotic plants (*Syngonium podophyllum*, *Epipremnum* spec., *Monstera* spec.) which she had watered in jars already during her stay on Martinique. For transportation to Germany, she had wrapped the plants in wet filter paper and put them in plastic bags. Upon arrival in Germany, she immediately transferred them into a water bowl in her living room where she kept further exotic plants under subtropical conditions (ca 25 °C, 60–70% relative humidity). In early April, she detected the first mosquitoes flying around in that room, which she caught and killed, not aware of their origin. Only in late May, she realised larval development in the plant bowl where she estimated dozens of larvae to be present. She immediately discarded the water with the larvae in the sink but continued to detect adult mosquitoes in the living room until mid-June when she submitted several specimens to the German citizen science project ‘Mueckenatlas’ (www.mueckenatlas.de), a passive mosquito surveillance initiative established in 2012 [1]. Later, the traveller reported having disposed of about the same number of adult mosquitoes killed in her living room as she had kept and submitted. From the time of submission to the ‘Mueckenatlas’, no more mosquitoes were observed in the household.

### Entomological investigations

Two mosquitoes, captured on 22 June 2016 in the living room of the German traveller to Martinique (subsequently referred to as 'the submitter'), were submitted to one of the research groups running the ‘Mueckenatlas’ project, from a small town close to Jena, German federal state of Thuringia (central eastern Germany). They were morphologically identified according to the determination key by Becker et al. [2] with subsequent genetic confirmation by CO1 barcoding [3]. Upon inquiry, the submitter made available an additional 11 mosquito specimens that she had successively collected in the same room and had kept in the freezer since (freezing is suggested by the managers of the ‘Mueckenatlas’ for killing the mosquitoes without damage). The mosquitoes were transported to the laboratory on dry ice to avoid RNA degradation.

Although all windows of the affected household were equipped with insect screens, immediately after the identification of the submitted mosquitoes, a small-scale monitoring using a set of 20 ovitraps and four gravid *Aedes* traps (GATs) distributed in the garden around the house of the submitter and its closer surroundings was implemented according to the European Centre for Disease Prevention and Control (ECDC) guidelines for the surveillance of invasive mosquitoes [4]. The traps were operated for a period of eight weeks and checked once a week for eggs and adult mosquitoes. In addition, artificial water containers in the neighbourhood gardens and in the village’s small cemetery (distance ca 450 m beeline) were systematically examined for mosquito developmental stages once a week for the same time period. No evidence of *Ae. aegypti* presence could be found outside the submitter’s house during the monitoring.

### Laboratory investigations of mosquitoes and the submitter

Mosquito homogenisation was performed as recently described [5]. The suspensions were clarified by centrifugation (5,000 g for 1 min), and the supernatant was used for RNA extraction with a QIAamp viral RNA Mini Kit (Qiagen) according to the manufacturer’s protocol. RNA extraction from blood plasma samples taken from the submitter was performed using the same kit. The extracted RNAs from both the mosquitoes and the plasma samples were analysed with the RealStar Zika Virus RT-PCR Kit, RealStar dengue RT-PCR Kit and RealStar chikungunya RT-PCR Kit (Altona Diagnostics, Hamburg, Germany) according to the manufacturer’s protocol.

Immunofluorescence assays for Zika virus (ZIKV), dengue virus (DENV) and chikungunya virus (CHIKV) were performed on the submitter’s plasma samples as recently described [6].

Morphologically, all submitted mosquitoes were unambiguously identified as *Ae. aegypti*. Although not a validated identification method for *Ae. aegypti*, CO1 barcoding of the first two specimens (GenBank accession numbers: KY022526, KY022527) showed 100% sequence homology with this species when aligned to BOLD (Barcode of Life database: www.boldsystems.org) and GenBank (www.ncbi.nlm.nih.gov/genbank) entries.

All mosquitoes tested negative for ZIKV, DENV and CHIKV RNA, and there was no serological or molecular evidence that the submitter had an acute or recent infection with any of these viruses.

## Discussion

*Ae. aegypti* (Linnaeus, 1762) is considered the most important culicid vector of viruses worldwide. Among the viruses transmitted by this species are yellow fever virus, DENV and ZIKV [7,8].

*Ae. aegypti* is a particularly thermophilic mosquito species, endemic in tropical and subtropical regions [9]. From the late 17th until the mid-20th century, it was also widely distributed in the Mediterranean, around the Black Sea and further on to the Caspian Sea. Numerous dengue and yellow fever epidemics with high fatality rates caused by this species are documented for Europe. Sporadically, during summer, populations also developed in more northern parts of Europe (e.g. France, United Kingdom) where they had been introduced by ships returning from the tropics [10]. The species had disappeared from Europe until the middle of the 20th century, but recently re-emerged on the eastern Black Sea coast, including southern Russia, Abkhasia, Georgia and eastern Turkey [11-13], and on the Portuguese Island of Madeira [14]. Introductions of mosquito eggs by the used tyre trade and of adult mosquitoes by aircraft have recently been reported from the Netherlands [15,16].

In the present ZIKV epidemic associated with congenital malformations in newborns in South and Central America, *Ae. aegypti* is considered the primary vector [17]. In addition, *Ae. aegypti* was incriminated as vector during the dengue fever outbreak in 2012 on the Island of Madeira [18].

The event described here (development of *Ae. aegypti* in Germany, although indoors, following importation of eggs attached to tropical plants) is of note for several reasons. First of all, the mosquito eggs were introduced from a region with an ongoing ZIKV epidemic that is endemic also for DENV and experienced a CHIKV outbreak in 2014, and it has been shown that all three viruses can be transmitted transovarially by *Ae. aegypti* [19-21]. However, this route of virus maintenance and propagation is probably very inefficient and epidemiologically irrelevant. Hence, the risk for the people in the household was limited. Second, the daughter of the traveller, who frequently visited the mosquito-infested household was pregnant during the infestation period (late first and early second trimester), and thus, her fetus could have been at risk in case of a congenital ZIKV infection. Notwithstanding, she did not consent to blood tests, neither did her brother and her father, because none of the family members had noticed mosquito bites during the period of infestation. Third, the case recalls the question whether *Ae. aegypti* is able to establish in central Europe. Most critical for the latter is probably the ability to overwinter. Eggs of *Ae. aegypti* are not resistant to freezing. However, in some states of the United States where winter temperatures may drop below ‑20 °C, local *Ae. aegypti* appear to have survived in sheltered sites, and theoretically this could also happen in Europe [22].

In conclusion, travel and trade lead to invasive mosquitoes being introduced from all over the world to non-endemic areas where they have the potential to reproduce and establish. The event presented here should raise awareness regarding potential introduction and possible establishment of invasive mosquito vectors through pathways other than the known commercial activities. As some mosquito species are vectors of disease agents and might even carry those already when introduced, implementation of appropriate surveillance schemes is becoming more and more important. The German passive monitoring instrument ‘Mueckenatlas’ has once more demonstrated its effectiveness as an early warning system.
